# Molecular epidemiologic tools for waterborne pathogens *Cryptosporidium* spp. and *Giardia duodenalis*

**DOI:** 10.1016/j.fawpar.2017.09.002

**Published:** 2017-09-29

**Authors:** Lihua Xiao, Yaoyu Feng

**Affiliations:** a Division of Foodborne, Waterborne, and Environmental Diseases, National Center for Emerging and Zoonotic Infectious Diseases, Centers for Disease Control and Prevention, Atlanta, GA 30329, USA; b College of Veterinary Medicine, South China Agricultural University, Guangzhou 510642, China

**Keywords:** *Cryptosporidium*, *Giardia*, Molecular epidemiology, Genotyping

## Abstract

Molecular diagnostic tools have played an important role in improving our understanding of the transmission of *Cryptosporidium* spp. and *Giardia duodenalis*, which are two of the most important waterborne parasites in industrialized nations. Genotyping tools are frequently used in the identification of host-adapted *Cryptosporidium* species and *G. duodenalis* assemblages, allowing the assessment of infection sources in humans and public health potential of parasites found in animals and the environment. In contrast, subtyping tools are more often used in case linkages, advanced tracking of infections sources, and assessment of disease burdens attributable to anthroponotic and zoonotic transmission. More recently, multilocus typing tools have been developed for population genetic characterizations of transmission dynamics and delineation of mechanisms for the emergence of virulent subtypes. With the recent development in next generation sequencing techniques, whole genome sequencing and comparative genomic analysis are increasingly used in characterizing *Cryptosporidium* spp. and *G. duodenalis*. The use of these tools in epidemiologic studies has identified significant differences in the transmission of *Cryptosporidium* spp. in humans between developing countries and industrialized nations, especially the role of zoonotic transmission in human infection. Geographic differences are also present in the distribution of *G. duodenalis* assemblages A and B in humans. In contrast, there is little evidence for widespread zoonotic transmission of giardiasis in both developing and industrialized countries. Differences in virulence have been identified among *Cryptosporidium* species and subtypes, and possibly between *G. duodenalis* assemblages A and B, and genetic recombination has been identified as one mechanism for the emergence of virulent *C. hominis* subtypes. These recent advances are providing insight into the epidemiology of waterborne protozoan parasites in both developing and developed countries.

## Highlights

• Molecular tools are widely used to characterize isolates of *Cryptosporidium* and *Giardia*.
• Methods differ in resolution capability, purpose, and technical complexity.
• Molecular characterization has significantly improved knowledge of cryptosporidiosis epidemiology.
• *Cryptosporidium* transmission is significantly different between developing and industrialized nations.
• Improved molecular tools are needed to delineate *Giardia duodenalis* transmission in humans.

## 1 Introduction

Protozoan parasites are important causes of diarrhea and other enteric diseases in humans (Custodio, 2016). They include mostly *Cryptosporidium* spp., *Giardia duodenalis*, *Cyclospora cayetanensis*, *Cystoisospora belli*, *Entamoeba histolytica*, and *Toxoplasma gondii*, although human infections with the latter are usually associated with non-gastrointestinal symptoms. Other protozoa in humans are not considered major pathogens, such as other *Entamoeba* spp., *Blastocystis hominis*, and *Balantidium coli*. As these pathogens use the fecal-oral route to maintain the lifecycle, foodborne and waterborne transmission are important in disease epidemiology (Thompson and Ash, 2016). Among them, *Cryptosporidium* spp. and *G. duodenalis* are best known for their potential to cause large waterborne outbreaks of illness (Efstratiou et al., 2017, Moreira and Bondelind, 2017), thus are major targets in recent research on waterborne protozoan pathogens in humans. Earlier waterborne outbreaks of diseases by these pathogens were mostly associated with drinking water, but more recently, recreational water-associated outbreaks of cryptosporidiosis and giardiasis are increasingly reported, and for cryptosporidiosis, they are now responsible for most waterborne outbreaks in the United States (Adam et al., 2016, Hlavsa et al., 2015, Painter et al., 2015).

Molecular diagnostic tools have long been used in studies of the transmission of *Cryptosporidium* spp. and *G. duodenalis* (Thompson and Ash, 2016, Xiao and Fayer, 2008). This is largely due to the existence of a large number of morphologically identical species or genotypes within both group of protozoa, which require the use of molecular diagnostic tools for differentiation. Thus far, over 30 *Cryptosporidium* species and many genotypes of unknown species status have been described (Ryan et al., 2014). Similarly, there are at least eight genotypes (assemblages) of *G. duodenalis* that are likely cryptic species (Feng and Xiao, 2011). Because of the existence of host specificity among *Cryptosporidium* species and *G. duodenalis* genotypes, characterizations of pathogens at the species or genotype level are helpful to the assessment of infection sources in humans and public health potential of parasites in animals and the environment. Advanced characterization of human-pathogenic species or genotypes can be used in case linkage, tracking of virulent subtypes, and assessment of disease burdens attributable to different transmission routes (Ryan and Caccio, 2013, Xiao, 2010). This can be achieved through conventional subtyping based on sequence analysis of individual polymorphic genes, multilocus typing, and comparative genomic analysis. In this report, we will review various molecular diagnostic tools used in the characterization of human-pathogenic *Cryptosporidium* spp. and *G. duodenalis*.

## 2 Genotyping tools

### 2.1 Genotyping tools for *Cryptosporidium* spp.

Accurate identification of *Cryptosporidium* species requires the use of genotyping tools. Currently, most *Cryptosporidium* genotyping tools use PCR targeting the small subunit (SSU) rRNA gene, largely because of the existence of conserved *Cryptosporidium*-specific sequence for designing primers that allow broad specific detection of all *Cryptosporidium* spp. and semi-conserved and hypervariable regions that can be used for the differentiation of various species and genotypes by restriction fragment length polymorphism (RFLP), melting curve, or DNA sequence analyses (Xiao, 2010). PCR tools targeting other genes were used in early *Cryptosporidium* research, but they are now infrequently used in *Cryptosporidium* genotyping because of their narrow detection range; they can only be used for genotyping *Cryptosporidium* species that are closely related to *C. parvum* or *C. hominis* because of the nature of sequences used in primer design (Jiang and Xiao, 2003).

Among the SSU rRNA-based *Cryptosporidium* genotyping tools, the PCR-RFLP tool using nested PCR amplification of a ~ 830-bp fragment and restriction analysis of the secondary PCR products using enzymes *Ssp*I and *Vsp*I (Xiao et al., 1999) is the most commonly used one. It can be used in genotyping *Cryptosporidium* spp. from both humans and animals. More recently, the use of *Vsp*I has been replaced with *Mbo*II in RFLP analysis of PCR products from *Cryptosporidium* spp. in ruminants (Feng et al., 2007). The technique requires stringent PCR conditions, but when optimized, can be used effectively in detecting single oocysts in water samples (Jiang et al., 2005, Xiao et al., 2006). Alternatively, a PCR assay that targeting a smaller fragment of the SSU rRNA gene can be used (Ryan et al., 2003). The technique is more adaptable to other PCR buffers, although is slightly less specific for *Cryptosporidium* (mostly presenting a minor problem to the analysis of water samples) and requires DNA sequence analysis in *Cryptosporidium* genotype determination.

In recent years, qPCR assays are increasingly used in genotyping human-pathogenic *Cryptosporidium* spp. Several species-specific genotyping tools based on the SSU rRNA and other genes have been developed for *C. hominis*, *C. parvum*, and *C. cuniculus* (Bouzid et al., 2016, Burnet et al., 2012, Hadfield and Chalmers, 2012, Hadfield et al., 2011, Jothikumar et al., 2008, Mary et al., 2013, Staggs et al., 2013, Yang et al., 2013). One major issue is the broad range of *Cryptosporidium* species that can occur in humans, which has limited the wide use of these species-specific qPCR assays. However, some of these tools also include a SSU rRNA-based generic qPCR assay for the detection of all *Cryptosporidium* species, which can be used in conjunction with *C. parvum* and *C. hominis*-specific assays for rapid differentiation of the two dominant *Cryptosporidium* species in human specimens. Recently, using fluorescence resonance energy transfer probes and melt curve analysis, one SSU rRNA-based PCR assays has been developed for rapid genotyping to five common *Cryptosporidium* species in human specimens (Li et al., 2015). In addition to the potential of streamlining the detection and identification of *Cryptosporidium* spp. in human clinical specimens, qPCR-based genotyping assays have been used in quantifying *Cryptosporidium* oocysts in these specimens (Mary et al., 2013, Operario et al., 2015, Yang et al., 2013), although low levels of oocysts that are typically found in drinking water samples cannot be accurately quantified (Staggs et al., 2013).

Next generation sequencing techniques are now increasingly used in *Cryptosporidium* genotyping, especially accurate identifications of mixed *Cryptosporidium* genotypes. Their usage in sequencing SSU rRNA and actin PCR products from several *Cryptosporidium* species of humans and animals was effectively demonstrated (Paparini et al., 2015). Although they are more expensive than traditional Sanger sequencing when analyzing small numbers of specimens, with the use of product indexing, the costs are comparable when specimens are processed in batches. With standardization, these techniques are likely to be used in routine *Cryptosporidium* genotyping in future (Ryan et al., 2017).

The use of genotyping tools in the analysis of clinical specimens has led to the identification of over 20 *Cryptosporidium* species and genotypes in humans (Table 1). With the exception of *C. meleagridis*, which infects both birds and mammals, all other human-pathogenic *Cryptosporidium* species and genotypes are pathogens of mammals. It is likely that some other mammalian *Cryptosporidium* species and genotypes will be found in humans in future. Clearly, these species and genotypes differ significantly in human infectivity, reflected by the differences in their frequency in both sporadic human cases and outbreaks. Molecular epidemiologic studies have also identified differences in the distribution of *Cryptosporidium* species and genotypes in humans between developing countries and industrialized nations. Thus, *C. hominis* is the dominant species in humans in developing countries, whereas both *C. hominis* and *C. parvum* are common in humans in most industrialized nations. Other species, such as *C. meleagridis*, *C. felis*, *C. canis*, *C. ubiquitum*, *C. cuniculus*, *C. muris* and chipmunk genotype I are mainly animal pathogens, with the exception of *C. viatorum*, which appears to be exclusively a human pathogen. These species are responsible for variable numbers of human infections depending on socioeconomic development and intensity of agriculture. Among them, *C. meleagridis*, *C. canis*, *C. viatorum* and *C. muris* are mostly found in children and HIV + persons in developing countries, whereas *C. ubiquitum*, *C. cuniculus*, and chipmunk genotype I are mainly found in immunocompetent and immunocompromised persons in industrialized nations. As these *Cryptosporidium* species and genotypes have different host ranges, the difference in *Cryptosporidium* species distribution could be a reflection of differences in the importance of various transmission routes in cryptosporidiosis epidemiology (Xiao, 2010). The remaining *Cryptosporidium* species or genotypes have been reported in only a few patients (Table 1). In addition to differences in human infectivity, *Cryptosporidium* species differ from each other in virulence, with *C. hominis* more virulent and causing more clinical presentations in most studies (Adamu et al., 2014, Cama et al., 2008, Cama et al., 2007, Dey et al., 2016, Iqbal et al., 2011). In a few studies, however, patients infected *C. parvum* had longer duration of diarrhea or higher occurrence of vomiting than those with *C. hominis*, (Eibach et al., 2015, Insulander et al., 2013).

**Table 1:** Human-pathogenic *Cryptosporidium* species and genotypes.

Species/genotype	Major host	No. cases in human	Reference
*C. hominis*	Humans, horses	Most common species	(Caccio and Chalmers, 2016, Squire and Ryan, 2017, Xiao, 2010, Xiao and Feng, 2008)
*C. parvum*	Ruminants	Common species	(Caccio and Chalmers, 2016, Squire and Ryan, 2017, Xiao, 2010, Xiao and Feng, 2008)
*C. meleagridis*	Galliformes	Numerous cases	(Elwin et al., 2012a, Insulander et al., 2013, Leoni et al., 2006, Stensvold et al., 2014, Wang et al., 2014b, Xiao and Feng, 2008)
*C. felis*	Cats	Numerous cases	(Lucio-Forster et al., 2010, Xiao and Feng, 2008)
*C. canis*	Dogs	Numerous reports	(Lucio-Forster et al., 2010, Xiao and Feng, 2008)
*C. ubiquitum*	Small ruminants, rodents, primates	59	(Cieloszyk et al., 2012, Elwin et al., 2012a, Fayer et al., 2010, Li et al., 2014, Moore et al., 2016, Network, 2010)
*C. cuniculus*	Rabbits	47	(Chalmers et al., 2011a, Chalmers et al., 2009b, Elwin et al., 2012a, Hadfield and Chalmers, 2012, Koehler et al., 2014, Molloy et al., 2010, Network, 2010, Puleston et al., 2014)
*C. viatorum*	Humans	32	(Adamu et al., 2014, Ayinmode et al., 2014, de Lucio et al., 2016, Elwin et al., 2012b, Insulander et al., 2013, Lebbad et al., 2013, Sanchez et al., 2017, Stensvold et al., 2015, Ukwah et al., 2017)
Chipmunk genotype I	Chipmunks, grey squirrels, deer mice	28	(Feltus et al., 2006, Insulander et al., 2013, Lebbad et al., 2013, Network, 2010)
*C. muris*	Rodents	18	(Al-Brikan et al., 2008, Azami et al., 2007, Gatei et al., 2002, Gatei et al., 2003, Gatei et al., 2006b, Hasajova et al., 2014, Neira et al., 2012, Palmer et al., 2003, Petrincova et al., 2015, Tiangtip and Jongwutiwes, 2002)
*C. andersoni*	Cattle and other bovine animals	7?^a^	(Agholi et al., 2013, Guyot et al., 2001, Hussain et al., 2017, Jiang et al., 2014, Leoni et al., 2006, Liu et al., 2014, Morse et al., 2007, Waldron et al., 2011)
*C. suis*	Pigs	5	(Bodager et al., 2015, Cama et al., 2007, Leoni et al., 2006, Moore et al., 2016, Xiao et al., 2002)
*C. bovis*	Cattle and other bovine animals	4	(Helmy et al., 2013, Khan et al., 2010, Ng et al., 2012)
Horse genotype	Horses	4	(Chalmers et al., 2009a, Robinson et al., 2008, Xiao et al., 2009)
*C. xiaoi*	Sheep and goats	2	(Adamu et al., 2014)
Skunk genotype	Rodents	2	(Chalmers et al., 2009a, Davies et al., 2009, Elwin et al., 2012a, Robinson et al., 2008)
Mink genotype	Minks, otters, ermines	2	(Ebner et al., 2015, Ng-Hublin et al., 2013)
*C. erinacei*	Hedgehogs	1	(Kvac et al., 2014)
*C. fayeri*	Marsupials	1	(Waldron et al., 2010, Waldron et al., 2011)
*C. scrofarum*	Pigs	1	(Kvac et al., 2009)
*C. tyzzeri*	Mice	1	(Raskova et al., 2013)

a Excluding data from three questionable reports.

### 2.2 Genotyping tools for *G. duodenalis*

Genotyping tools have been widely used in studies of the transmission of *G. duodenalis* in humans and animals (Thompson and Ash, 2016). This has led to the identification of eight genotypes or assemblages of *G. duodenalis* that differ from each other in host range. Among them, assemblages A and B are major human-pathogens, but are also found in various mammals. In contrast, assemblages C to H are host-adapted *G. duodenalis* genotypes, being found in specific groups of animals (Table 2). Therefore, assemblages C and D are mostly found in canine animals, assemblage E in hoofed livestock and wildlife, assemblage F in cats, assemblage G in rodents, and assemblage H in seals and other marine mammals.

**Table 2:** *Giardia duodenalis* assemblages in mammals and their host range.

Assemblage	Major host	No. cases in human	Reference
A	Humans, non-human primates, domestic and wild ruminants, alpacas, pigs, horses, domestic and wild canines, cats, ferrets, rodents, marsupials, other mammals	Major assemblage in humans	(Feng and Xiao, 2011, Ryan and Caccio, 2013, Squire and Ryan, 2017)
B	Humans, non-human primates, horses, rabbits, beavers, muskrats, chinchillas	Major assemblage in humans	(Feng and Xiao, 2011, Ryan and Caccio, 2013, Squire and Ryan, 2017)
C^a^	Dogs and other canine animals	8^c^	(Durigan et al., 2017, Soliman et al., 2011, Sprong et al., 2009)
D^a^	Dogs and other canine animals	6	(Broglia et al., 2013, Sprong et al., 2009)
E^a^	Ruminants, pigs, horses	59^b^	(Abdel-Moein and Saeed, 2016, Fantinatti et al., 2016, Foronda et al., 2008, Helmy et al., 2014, Scalia et al., 2016, Sprong et al., 2009, Zahedi et al., 2017)
F^a^	Cats	7	(Gelanew et al., 2007, Sprong et al., 2009)
G	Mice, rats	0	
H	Seals	0	

a Excluding specimens identified at the SSU rRNA locus.

b Including 26 from one study in Egypt (Abdel-Moein and Saeed, 2016), 20 from two studies in Brazil (Fantinatti et al., 2016, Scalia et al., 2016), and 6 from one study in Australia (Zahedi et al., 2017).

c Excluding 16 from one questionable study in China (Liu et al., 2014).

Early genotyping of *G. duodenalis* had used mostly PCR-sequence analysis of the SSU rRNA gene. Because of the low sequence polymorphism at this genetic locus, the use of these tools have led to the erroneous detections of *G. duodenalis* assemblages C, D, E and F in a small number of human specimens (Feng and Xiao, 2011). More recent genotyping assays employ PCR targeting the more polymorphic β-giardin (*bg*), glutamate dehydrogenase (*gdh*), and triosephosphate isomerase (*tpi*) genes (Ryan and Caccio, 2013). Because differences in the sensitivity of the PCR assays and occasional discrepant genotyping results among genetic loci, it is recommended that *G. duodenalis* genotypes be determined by multilocus analysis (Caccio et al., 2008), although human specimens genotyped at the three markers occasionally cannot be assigned unequivocally to either assemblage A or B (Broglia et al., 2013).

DNA sequence analysis of PCR products are generally used in determination of *G. duodenalis* genotypes. However, RFLP analysis of PCR products is still used in resource-limited countries (Colli et al., 2015, El Basha et al., 2016, Faria et al., 2016, Mbae et al., 2016, Skhal et al., 2017). This approach needs validation, as intragenotypic nucleotide variations in these polymorphic genes are common and are generally dispersed across the entire genes, leading to possible variations in RFLP patterns within some *G. duodenalis* assemblages such as assemblage B. In recent years, assemblage-specific PCR and qPCR assays have been developed for the detection of *G. duodenalis* assemblages A, B and E in clinical specimens (Lebbad et al., 2011, Van Lith et al., 2015, Vanni et al., 2012). These assays are valuable in the detection of concurrent infections with mixed *G. duodenalis* genotypes (Van Lith et al., 2015).

The use of genotyping tools have identified mostly the presence of *G. duodenalis* assemblages A and B in humans (Feng and Xiao, 2011, Ryan and Caccio, 2013, Squire and Ryan, 2017). Globally, more humans are infected with assemblage B than assemblage A. This is especially the case for Asian, Oceanian, European, and African countries (Feng and Xiao, 2011, Ryan and Caccio, 2013, Squire and Ryan, 2017). Both genotypes appear to be equally distributed in the Americas and human infections in Mexico are largely caused by assemblage A (Feng and Xiao, 2011, Ryan and Caccio, 2013).

Other *G. duodenalis* genotypes have been only occasionally detected in humans, with the possible exception of assemblage E, which has recently been detected in substantial numbers of human cases in rural areas of Egypt, Rio de Janeiro, Brazil, and Queensland, Australia (Abdel-Moein and Saeed, 2016, Fantinatti et al., 2016, Zahedi et al., 2017). In contrast, animals are mostly infected with host-adapted *G. duodenalis* assemblages (C and D in canine animals, E in hoofed livestock, F in cats, and G in rodents) and assemblage A, with noticeable exception of nonhuman primates, beavers and a few other animals, which are commonly infected with assemblage B (Feng and Xiao, 2011). The different distribution of *G. duodenalis* assemblages between humans and animals indicated that unlike in cryptosporidiosis, zoonotic transmission might not be important in giardiasis epidemiology.

Epidemiological studies of giardiasis in humans using genotyping tools have also revealed possible virulence differences between *G. duodenalis* assemblages A and B in humans. Some reports suggest that assemblage A is more virulent than assemblage B (Aydin et al., 2004, Breathnach et al., 2010, Haque et al., 2005, Hussein et al., 2016, Perez Cordon et al., 2008, Read et al., 2002, Sahagun et al., 2008), while others, especially several recent ones, have indicated the opposite (Al-Mohammed, 2011, Fahmy et al., 2015, Faria et al., 2017b, Gelanew et al., 2007, Homan and Mank, 2001, Lebbad et al., 2011, Minetti et al., 2015a, Mohammed Mahdy et al., 2009, Pelayo et al., 2008). One factor not considered was the intra-assemblage variation, which could possibly account for some differences between the studies (Emery et al., 2014, Monis et al., 2009). Data on genotype distribution of *G. duodenalis* in outbreaks of human giardiasis are helpful in delineating differences in virulence between assemblages A and B in humans, but only a dozen of outbreaks have been investigated thus far using genotyping tools. Most earlier reported giardiasis outbreaks were caused by assemblage B, with the exception of one waterborne outbreak in Canada and one hospital-associated outbreak in China, which were caused by assemblage A (Feng and Xiao, 2011, Figgatt et al., 2017, Wang et al., 2013), indicating that assemblage B could be more virulent than assemblage A. A recent report, however, has shown that all four drinking water-associated outbreaks of giardiasis in small towns, British Columbia investigated were caused by mixed assemblages A and B, with B dominating (Prystajecky et al., 2015). Despite the higher proportion of human infections due to assemblage B, assemblage A, especially the anthroponotic sub-assemblage AII, appears to be the dominant *G. duodenalis* in urban wastewater (Ben Ayed et al., 2012, Caccio et al., 2003, Castro-Hermida et al., 2008, Hatam-Nahavandi et al., 2017, Li et al., 2012, Liu et al., 2011, Ma et al., 2016, Ramo et al., 2017, Samie and Ntekele, 2014, Sulaiman et al., 2004, Ulloa-Stanojlovic et al., 2016). Thus, in addition to possible differences in virulence, there could be other biological differences between the two major human-pathogenic *G. duodenalis* assemblages.

## 3 Subtyping tools

### 3.1 Subtyping tools for *Cryptosporidium* spp.

Subtyping tools have been used extensively in assessing intra-species diversity of *C. parvum*, *C. hominis* and other human-pathogenic *Cryptosporidium* spp. in endemic areas, role of zoonotic infections in cryptosporidiosis epidemiology, and contamination or infection sources during cryptosporidiosis outbreaks. Most subtyping tools are based on sequence analysis of the gene encoding the 60 kDa glycoprotein (*gp60*), which is known to be involved in invasion by *Cryptosporidium* spp. (Strong et al., 2000). The *gp60* gene is highly polymorphic, categorizing *C. parvum* and *C. hominis* each into multiple subtype families by nucleotide sequence differences. Within each subtype family, subtypes differ from each other mostly in the number of tri-nucleotide repeats (TCA, TCG or TCT) region downstream of the signal peptide sequence of the gene (Strong et al., 2000).

A nomenclature system has been established for naming *Cryptosporidium* subtypes (Strong et al., 2000, Sulaiman et al., 2005, Xiao, 2010). The name of *Cryptosporidium* subtypes starts with the subtype family designation (Ia, Ib, Id, Ie, If, etc. for *C. hominis*; IIa, IIb, IIc, IId, etc. for *C. parvum*; IIIa, IIIb, IIIc, IIId, etc. for *C. meleagridis*). This is followed by the number of TCA (represented by the letter A), TCG (represented by the letter G), or TCT (represented by the letter T) repeats (Feng et al., 2011a, Sulaiman et al., 2005, Xiao, 2010). For example, the subtype name IeA11G3T3 indicates that isolate belongs to the *C. hominis* subtype family Ie and has 11 copies of the TCA repeat, 3 copies of the TCG repeat, and 3 copies of the TCT repeat in the trinucleotide repeat region of the gene. In some subtype families, isolates may have identical number of the trinucleotide repeats, but differ from each other in the copy number of other repetitive sequences (designed as R at the end of the subtype name). For example, in the *C. parvum* IIa subtype family, subtypes have 1–3 copies of the ACATCA sequence immediately downstream from the trinucleotide repeats, which are represented by R1, R2 and R3. Similarly, subtypes within the *C. hominis* subtype families Ia and If frequently have different copies of a 15-bp repetitive sequence (AAGACGGTGGTAAGG or its variations in the Ia subtype family and AAGAAGGCAAAGAAG or its variations in the If subtype family). In addition, subtypes in the *C. parvum* subtype family IIc all have five copies of TCA and three copies of TCG repeats (IIcA5G3), but differ from each other in sequences of the 3′ region of the gene. These subtypes are differentiated from each by alphabetical extensions, such as IIcA5G3a and IIcA5G3b. The GenBank accession numbers of known *Cryptosporidium* subtype families are shown in Table 3.

**Table 3:** Known *Cryptosporidium* subtype families based on sequences of the 60 kDa glycoprotein gene.

Species	Subtype family	Trinucleotide repeat	Other repeat (R)	GenBank accession no.**
*C. hominis*	Ia	TCA	AAGACGGTGGTAAGG	AF164502 (IaA23R4)
	Ib	TCA, TCG, TCT	–	AY262031 (IbA10G2)
				DQ665688 (IbA9G3)
	Id	TCA, TCG	–	DQ665692 (IdA16)
	Ie	TCA, TCG, TCT	–	AY738184 (IeA11G3T3)
	If	TCA, TCG	AAGAAGGCAAAGAAG	AF440638 (IfA19G1R5), FJ153244 (IfA22G1R4)
	Ig	TCA	–	EF208067 (IgA24)
	Ih	TCA, TCG	–	FJ971716 (IhA14G1)
	Ii	TCA	–	HM234173 (IiA17)
	Ij	TCA		JF681174 (IjA14)
	Ik			KJ941148 (IkA15G1)
*C. parvum*	IIa	TCA, TCG	ACATCA	AY262034 (IIaA15G2R1), DQ192501 (IIaA15G2R2)
	IIb	TCA	–	AF402285 (IIbA14)
	IIc	TCA, TCG	–	AF164491 (IIcA5G3a)
				AF164501 (IIcA5G3b)
				EU095267 (IIcA5G3c)
				AF440636 (IIcA5G3d)
				HM234172 (IIcA5G3e)
				AJ973154 (IIcA5G3h)
				AM947935 (IIcA5G3i)
				GQ259136 (IIcA5G3j)
				JF802123 (IIcA5G3k)
				KU670809 (IIcA5G3l)
				KU670810 (IIcA5G3m)
				KU670811 (IIcA5G3n)
				KU670812 (IIcA5G3o)
				JF268646, JN867336 (IIcA5G3p)
				KM539034 (IIcA5G3q)
				KF957656 (IIcA5G3r)
	IId	TCA, TCG	–	AY738194 (IIdA18G1)
	IIe	TCA, TCG	–	AY382675 (IIeA12G1)
	IIf	TCA	–	AY738188 (IIfA6)
	IIg	TCA	–	AY873780 (IIgA9)
	IIh	TCA, TCG	–	AY873781 (IIhA7G4)
	IIi	TCA	–	AY873782 (IIiA10)
	IIk	TCA	–	AB237137 (IIkA14)
	IIl	TCA	–	AM937006 (IIlA18)
	IIm	TCA, TCG		AY700401(IImA7G1)
	IIn	TCA		FJ897787 (IInA8)
	IIo	TCA, TCG		JN867335 (IIoA16G1)
	IIp	TCA		KC885904 (IIpA9)
	IIq	TCA		KU670813 (IIqA6R2)
	IIr	TCA, TCG		KU852719 (IIrA5G1)
	IIs	TCA, TCG		KU852720 (IIsA14G1)
	IIt	TCA		KU852718 (IItA13R1)
*C. meleagridis*	IIIa	TCA, TCG	–	AF401499 (IIIaA24G3)
	IIIb	TCA, TCG	–	AB539720 (IIIbA20G1)
	IIIc	TCA	–	AF401497 (IIIcA6)
	IIId	TCA	–	DQ067570 (IIIdA6)
	IIIe	TCA, TCG	–	AB539721 (IIIeA20G1)
	IIIf	TCA, TCG		EU164813 (IIIfA16G2)
	IIIg	TCA, TCG		JX878614 (IIIgA19G5)
	IIIh	TCA		KU831548 (IIIhA7)
	IIIi	TCA		KP730324 (IIIiA13)
	IIIj	TCA		KP730324 (partial at 5′)
*C. fayeri*	IVa	TCA, TCG, TCT	–	FJ490060 (IVaA11G3T1)
	IVb	TCA, TCG, TCT	–	FJ490087 (IVbA9G1T1)
	IVc	TCA, TCG, TCT	–	FJ490069 (IVcA8G1T1)
	IVd	TCA, TCG, TCT		FJ490058 (IVdA7G1T1)
	IVe	TCA, TCG, TCT	–	FJ490071(IVeA7G1T1)
	IVf	TCA, TCG, TCT	–	FJ490076 (IVfA12G1T1)
Opossum genotype	XIa	TCA, TCG, TCT	–	HM234181 (XIaA4G1T1)
*C. cuniculus*	Va	TCA	–	FJ262730 (VaA18)
	Vb	TCA	–	FJ262734 (VbA29)
Horse genotype	VIa	TCA, TCG	–	FJ435960 (VIaA11G3), DQ648547 (IIjA15G4)
	VIb	TCA		FJ435961 (VIbA13)
	VIc	TCA		KU852738 (VIcA16)
*C. wrairi*	VIIa	TCA, TCT	–	GQ121020 (VIIaA17T1)
Ferret genotype	VIIIa	TCA, TCG	–	GQ121029 (VIIIaA5G2)
*C. tyzzeri*	IXa	TCA	ATTCTGGTACTGAAGATA	GQ121030 (IXaA6R3), AY738188 (IXaA6R2), HM234177 (IXaA6R2)
	IXb	TCA	–	HM234176 (IXbA6)
Mink genotype	Xa	TCA, TCG	–	HM234174 (XaA5G1)
Opossum genotype I	XIa	TCA, TCG, TCT		HM234181 (XIaA4G1T1)
*C. ubiquitum*	XIIa	–		JX412915
	XIIb	–		JX412926
	XIIc	–		JX412925
	XIId	–		JX412922
	XIIe	–		KC204983
	XIIf	–		KC204984
*C. erinaci*	XIIIa	TCA	ACATCA	KF055453 (XIIIaA20R10)
Chipmunk genotype I	XIVa	TCA, TCG, TCT		KP099082 (XIVaA18G2T1a), KP099086 (XIVaA18G2T1b), KP099085 (XIVa19G2T2a), KP099084 (XIVa19G2T2b), KP099083 (XIVaA20G2T2)
*C. viatorum*	XVa	TCA		KP115936 (XVaA3a), KP115937 (XVaA3b), KP115938 (XVaA3c), KP115939 (XVaA3d), KP115940 (XVaA3e), KP115941 (XVaA3f)
Skunk genotype	XVIa	TCA		KP099095 (XVIa14a)

Subtyping tools targeting the *gp60* gene has been developed recently for several other human-pathogenic *Cryptosporidium* species such as *C. meleagridis*, *C. ubiquitum*, *C. viatorum*, and *Cryptosporidium* skunk genotype and chipmunk genotype I (Guo et al., 2015a, Li et al., 2014, Stensvold et al., 2014, Stensvold et al., 2015, Yan et al., 2017). These new subtyping tools are needed as PCR primers based on *C. parvum* and *C. hominis* sequences cannot amplify efficiently the *gp60* gene of these divergent *Cryptosporidium* spp. Recent whole genome sequencing has greatly facilitated the design of PCR primers for them. Subtyping tools for other important zoonotic *Cryptosporidium* species such as *C. felis* and *C. canis* are not yet available due to the lack of whole genome sequence data.

There are significant phenotypic differences among subtype families of *Cryptosporidium* spp. Among the three most common *C. parvum* subtype families, IIa is mostly found in cattle, IId is mostly found in sheep and goats, whereas IIc is mostly found in humans, with the former two responsible for zoonotic cryptosporidiosis (Xiao, 2010). However, in areas with extensive transmission of *C. parvum* in cattle, such as European countries, IIa subtypes can be found in lambs and goat kids, while in China, where IIa subtypes are largely absent in the country, dairy calves are mostly infected with IId subtypes. Host adaptation has also been identified in *C. ubiquitum* and *C. tyzzeri* based on sequence analysis of the *gp60* gene (Kvac et al., 2013, Li et al., 2014). In humans, differences in clinical presentations and virulence have been observed among some *C. hominis* or *C. parvum* subtype families (Cama et al., 2008, Cama et al., 2007, Del Chierico et al., 2011, Feng et al., 2012, Iqbal et al., 2011). At the subtype level, *gp60* subtypes IbA10G2 of *C. hominis* and IIaA15G2R1 of *C. parvum* are widely distributed and responsible for numerous outbreaks of human cryptosporidiosis, probably due to their biological fitness and high virulence (Chalmers and Caccio, 2016, Feng et al., 2013, Li et al., 2013). In Peru, this subtype is associated with more clinical symptoms in infected children than other *C. hominis* subtypes (Cama et al., 2008).

### 3.2 Subtyping tools for *G. duodenalis*

Subtyping of *G. duodenalis* is currently done by sequence analysis of the PCR products of *tpi*, *gdh*, and *bg* genes, which leads to the identification of subtypes in addition to assemblages, based on the presence of SNPs within each assemblage (Feng and Xiao, 2011). Within assemblage A, there are several subtypes at each of the genetic loci, frequently named as A1–A6. At the *gdh* locus, A1 and A5 subtypes have similar sequences, forming a subgroup that is divergent from the second subgroup formed by A2-A4. In contrast, the A6 subtype has very divergent sequence from other subtypes. The separation of the former two subgroups is not as evident at the *tpi* and *bg* loci, although the A6 subtype has very divergent sequences at both loci (Feng and Xiao, 2011). There is apparent host adaption at the subtype level for the three common assemblage A subtypes at each genetic locus, with A1 subtype mostly found in animals, A2 mostly in humans, and A6 almost exclusively in wild ruminants (Feng and Xiao, 2011).

Extensive genetic heterogeneity also occurs in assemblage B and other *G. duodenalis* genotypes. There are many more subtypes of assemblage B at these three genetic loci than assemblage A subtypes, but there are no robust formations of subgroups of assemblage B subtypes in phylogenetic analysis of *tpi*, *gdh*, and *bg* sequences (Feng and Xiao, 2011). Indeed because of the presence of allelic sequence heterozygosity in assemblage B at these genotyping and subtyping loci (Ankarklev et al., 2012, Caccio et al., 2008), it may be difficult to subtype assemblage B specimens based on sequence analysis of PCR products from these loci (Caccio and Ryan, 2008). Many subtypes are also seen in assemblage E, with no formation of host-specific subgroups in nucleotide sequences from the three genetic loci. Subtyping of assemblage E specimens has been used extensively in recent studies of *G. duodenalis* transmission in ruminants in China (Qi et al., 2016a, Wang et al., 2016a, Wang et al., 2017, Wang et al., 2016b, Zhao et al., 2015).

## 4 Multilocus typing tools

### 4.1 Multilocus typing tools for *Cryptosporidium* spp.

Because of the likely occurrence of genetic recombination among isolates, results of *gp60*-based subtyping alone are unlikely to reflect intra-species diversity of *Cryptosporidium* spp. in disease endemic areas (Widmer and Lee, 2010). To increase the resolution of *gp60*-based subtyping and facilitate population genetic studies, multilocus fragment (MLFT) or sequence (MLST) typing tools have been developed for *C. parvum* and *C. hominis* (Gatei et al., 2006a, Mallon et al., 2003b). These tools largely target sequences with microsatellite and minisatellite repeats, which are also known as variable-number tandem-repeats.

Results of isolate characterizations using MLFT tools have confirmed the existence of host-segregated *C. parvum* populations (Drumo et al., 2012, Mallon et al., 2003a, Quilez et al., 2013) and identified differences in population genetic structure between *C. parvum* and *C. hominis* (Mallon et al., 2003a, Mallon et al., 2003b). In addition, significant geographic segregation of *C. parvum* populations has been identified among European countries, with population isolation by physical distance (Caccio et al., 2015). However, because of a predominantly panmictic population structure, there has been no apparent geographic sub-structuring in *C. parvum* within Scotland and the Upper Midwest United States (Herges et al., 2012, Mallon et al., 2003a, Mallon et al., 2003b, Morrison et al., 2008). As a result, distinct *C. parvum* MLFT types are frequently confined to individual farms (Quilez et al., 2011, Tanriverdi et al., 2006). In Spain, *C. parvum* in cattle has a panmictic structure while *C. parvum* in sheep has a clonal structure, probably a reflection of differences in population genetic structure between *gp60* IIa and IId subtype families (Ramo et al., 2016a, Ramo et al., 2016b). Recently, efforts have been made to evaluate existing MLFT markers (Chalmers et al., 2017, Hotchkiss et al., 2015) and identify additional markers for robust MLFT analysis of *C. parvum* specimens (Perez-Cordon et al., 2016).

MLST tools are increasingly used in genetic characterizations of *C. parvum* and *C. hominis* specimens from humans and animals (Feng et al., 2014, Feng et al., 2013, Gatei et al., 2008, Gatei et al., 2007, Gatei et al., 2006a, Li et al., 2013, Wang et al., 2014a, Yadav et al., 2017). Compared with MLFT, MLST tools have the advantage of unambiguous comparability of nucleotide sequence data among distant laboratories and possible interpretation of MLST data in the context of whole genome sequence data (for example, it is possible to infer MLST types based on whole genome sequences for comparative analysis of existing data). The use of these tools in the characterizations of specimens from endemic areas has identified mostly a clonal population structure for *C. hominis* (Gatei et al., 2007) and the existence of geographically segregated *C. hominis* subpopulations among developing countries, with populations in Jamaica and Peru significantly different from those in India and Kenya (Gatei et al., 2008, Gatei et al., 2006a). It has further supported the clonal nature of *C. parvum* IId subtypes from diverse areas (Wang et al., 2014a). A systematic MLST analysis of 32 polymorphic markers in chromosome 6 had shown a high genetic heterogeneity within *C. hominis* IbA10G2 specimens from children living in a small community in Lima, Peru. Despite the overall clonal population structure of *C. hominis* subtypes in the community, genetic recombination was detected in the virulent IbA10G2 subtype (Li et al., 2013). Genetic recombination was also shown to be a driving force for the emergence of the major *C. hominis* outbreak subtype IaA28R4 and *C. parvum* outbreak subtype IIaA15G2R1 in the United States (Feng et al., 2014, Feng et al., 2013).

One of the MLST tools has been used effectively in the identification of a clonal population structure of *C. meleagridis* and the likely occurrence of cross-species transmission of infections between birds and human in Lima, Peru (Wang et al., 2014b). Based on some of the same targets and additional ones from comparative genomic analysis, a MLST tool has been developed for *C. ubiquitum*, which has confirmed the existence of host-adapted *C. ubiquitum* populations and likely occurrence of genetic recombination among rodent-adapted *gp60* subtype families (Tang et al., 2016). An MLST tool has also been developed for the gastric species *C. andersoni* and *C. muris* (Feng et al., 2011b). It has been used effectively in population genetic characterizations of bovine *C. andersoni* in China (Qi et al., 2016b, Wang et al., 2012, Zhao et al., 2013, Zhao et al., 2014), with the identification of mostly an epidemic population structure (Qi et al., 2016b, Wang et al., 2012).

### 4.2 Multilocus typing tools for *G. duodenalis*

There are currently no high-resolution MLST tools for *G. duodenalis*. The multilocus genotyping tool based on sequence analysis of the *gdh*, *bg*, and *tpi* loci has been used as a MLST tool in subtyping assemblages A, B and E as discussed above. Data from these loci have even been used in population genetic characterizations of the pathogen in humans and animals (Choy et al., 2015, Durigan et al., 2017, Gabin-Garcia et al., 2017). An MLST subtype nomenclature system has been developed for assemblage A based on sequence analysis of the *bg*, *gdh*, and *tpi* genes (Caccio et al., 2008). This has allowed the identification of three sub-assemblages within assemblage A: AI, AII, and AIII (Table 4). Similar to the existence of host-adaptation at the subtype level, there are differences in the distribution of these sub-assemblages among hosts, with AI dominating in animals, AII dominating in humans, and AIII mostly in wild ruminants, although the former two are both human pathogens (Feng and Xiao, 2011).

**Table 4:** *Giardia duodenalis* subtypes at the glutamate dehydrogenase (*gdh*), β-giardin (*bg*), and triosephosphate isomerase (*tpi*) loci^a^.

Sub-assemblage	MLG type	Subtype			GenBank accession number			Major host
		gdh	bg	tpi	gdh	bg	tpi	
AI	AI-1	A1	A1	A1	AY178735, EF507606, EF685701, EF507610	X14185, AY258617, EU769204, X85958	L02120, AY655704, AF069556, EF688040	Animals, humans
	AI-2	A5	A5	A5	M84604, EU362969, EF507598	AB469365, DQ649780, DQ984131, AB218605	AB509383, EU781000, KP780972, JQ837805	Cats
AII	AII-1	A2	A2	A2	AY178737, EF507674, EU362964, L40510	AY072723, FJ971422, EU594669, FJ560582	U57897, KM190773, EF688019, AB569403	Humans, animals
	AII-2	A3	A3	A2	EU278608	AY072724, FJ971415, EU188635, FJ471821	U57897, KM190773, EF688019, AB569403	Humans
	AII-3	A3	A2	A2	EU278608	AY072723, FJ971422, EU594669, FJ560582	U57897, KM190773, EF688019, AB569403	Humans
	AII-4	A4	A3	A2	EF507657, EF507680, EF507651, EF507676	AY072724, FJ971415, EU188635, FJ471821	U57897, KM190773, EF688019, AB569403	Humans
	AII-5	A3	A3	A1	EU278608	AY072724, FJ971415, EU188635, FJ471821	L02120, AY655704, AF069556, EF688040	Humans
	AII-6	A3	A3	A3	EU278608	AY072724, FJ971415, EU188635, FJ471821	EU041754	Humans
	AII-7	A3	A3	A4	EU278608	AY072724, FJ971415, EU188635, FJ471821	GQ329677, AB509382, EU781027, EU637593	Humans
	AII-8	A4	A2	A2	EF507657, EF507680, EF507651, EF507676	AY072723, FJ971422, EU594669, FJ560582	U57897, KM190773, EF688019, AB569403	Humans
	AII-9	A2	A3	A2	AY178737, EF507674, EU362964, L40510	AY072724, FJ971415, EU188635, FJ471821	U57897, KM190773, EF688019, AB569403	Humans
AIII	AIII-1	A6	A6	A6	EU637582, DQ100288, HM150751, KT270859	DQ648777, DQ650649, EU216429, EU621373	DQ650648, EU781002, HM150750, KU531707	Wild ruminants

a Based on (Feng and Xiao, 2011), with the addition of AII-8 and AII-9 (Faria et al., 2017a).

In contrast, sequence analysis of assemblage B specimens at the three genetic loci has identified numerous subtypes, which are inconsistently segregated among loci. Thus, the BIII and BIV sub-assemblages originally identified by allozyme analysis are not supported by MLST analysis (Feng and Xiao, 2011). MLST analysis of assemblage B specimens is further complicated by allelic sequence heterozygosity at some of the genetic loci. This also occurs in assemblages C, D and E, but not assemblage A at these genotyping loci (Lebbad et al., 2010). Nevertheless, geographic segregation and host-adaptation have been identified in assemblage B in captive nonhuman primates in China using the current MLST tool; isolates from China were significantly different from those from Italy and Sweden, and isolates from ring-tailed lemurs and squirrel monkeys were genetically different from those from other nonhuman primates (Karim et al., 2015). Clearly, better MLST tools targeting genetic loci with homogenous sequences are needed for high-resolution typing and population genetic characterizations of *G. duodenalis*. This becomes feasible with the recent identification of numerous highly polymorphic genes within assemblage A or B through comparative genomics (Ankarklev et al., 2015, Wielinga et al., 2015).

## 5 Comparative genomic analysis

### 5.1 Comparative genomic analysis of *Cryptosporidium* spp.

Comparative genomics is increasingly used in the characterization of human pathogenic *Cryptosporidium* spp. For a considerable period, whole genome sequence (WGS) data are available from only four laboratory-propagated *Cryptosporidium* isolates (Mazurie et al., 2013, Widmer and Sullivan, 2012). With the recent development of next generation sequencing techniques and procedures for isolation and enrichment of pure *Cryptosporidium* DNA (Andersson et al., 2015, Guo et al., 2015b, Hadfield et al., 2015, Troell et al., 2016), many *Cryptosporidium* isolates have been sequenced and data of them are available in public databases such as NCBI and CryptoDB (Feng et al., 2017, Guo et al., 2015c, Ifeonu et al., 2016, Isaza et al., 2015, Liu et al., 2016, Sikora et al., 2017). As expected, most of the isolates sequenced are from *C. parvum* and *C. hominis*, but several other *Cryptosporidium* species have also been sequenced, including *C. meleagridis*, *C. ubiquitum*, *C. baileyi*, and *C. andersoni* (Ifeonu et al., 2016, Liu et al., 2016).

Despite the high sequence similarity (~ 97%) between *C. parvum* and *C. hominis* genomes, comparative analyses of the WGS data have identified some differences in gene content between the two major human-pathogenic *Cryptosporidium* species. The gene gains and losses mainly occur in the subtelomeric regions of several chromosomes, and involve mostly two *Cryptosporidium*-specific secretory protein families: MEDLE proteins and insulinase-like proteases (Guo et al., 2015c). These differences have also been observed between them and *C. ubiquitum*, and between *C. parvum gp60* subtype families IIa and IId (Feng et al., 2017, Liu et al., 2016). In addition to these gene gains and losses, these *Cryptosporidium* species and *C. parvum* have highly divergent sequences in genes encoding invasion-associated and immunodominant mucin proteins and other families of secretory proteins. These genetic differences could be responsible for some of the phenotypic differences among closely related *Cryptosporidium* species and host-adapted *C. parvum* subtype families (Feng et al., 2017, Guo et al., 2015c, Liu et al., 2016). This has been recently supported by a preliminary study of MEDLE-2, which was shown to be involved in the invasion of host cells (Li et al., 2017).

Comparative genomic analysis of *C. hominis* isolates from the United States has confirmed the occurrence of genetic recombination in virulent outbreak subtypes IbA10G2 and IaA28R4 (Guo et al., 2015c). Genomes of the two subtypes are highly similar, with major sequence differences occurring at the 5′ (around cgd6_60) and 3′ (around cgd6_5270) subtelomeric regions and around *gp60* (cgd6_1080), all within chromosome 6. In these areas, the genomes have shown some hallmarks of genetic recombination, with bi-allelic sequences in these areas and a mosaic pattern in sequence types across the chromosome. As the sequence differences involve a secretory protease (cgd6_60) and an invasion-related mucin protein (*gp60*), both of which are among the most polymorphic genes in *Cryptosporidium* spp. (Li et al., 2013, Tang et al., 2016), genetic recombination could play a potential role in the emergence of hyper-transmissible *C. hominis* subtypes, as previously indicated by MLST characterizations of IbA10G2 in Peru and IaA28R4 in the United States (Feng et al., 2014, Guo et al., 2015c).

The genomes of *C. hominis* IbA10G2 in European countries appear to be highly conserved (Chalmers and Caccio, 2016, Beser et al., 2017). A comparative genomic analysis of WGS data from several IbA10G2 isolates has identified fewer than 50 SNPs among most isolates from Europe, with the exception of one isolate (SWEH9) from Spain (Chalmers and Caccio, 2016, Sikora et al., 2017). In contrast, the genome of IbA10G2 isolates from Guatemala is divergent from genomes of European isolates (Sikora et al., 2017). This is in contrast with IbA10G2 in the United States, where two types of genomes are present among the few isolates sequenced, one of which is similar to the one in European countries (Guo et al., 2015c). Several SNP features unique to the *C. hominis* IbA10G2 subtype have been identified in a recent comparative genomic analysis, including the restoration of a loss-of-function mutation seen in other *C. hominis* subtypes from developing countries (Sikora et al., 2017). The high genome sequence conservation in IbA10G2 presents a significant challenge in epidemiologic tracking of this subtype, which is a major outbreak subtype in European countries and responsible for almost all autochthonous *C. hominis* infections there. Recently, based on WGS data from 17 isolates, a panels of nine loci with 10 SNVs were selected for the development of a PCR-next generation sequencing technique, which had identified 10 sequence types among 44 clinical isolates in Sweden (Beser et al., 2017).

### 5.2 Comparative genomic analysis of *G. duodenalis*

WGS data are available from several laboratory isolates of assemblages A, B and E (Adam et al., 2013, Franzen et al., 2009, Jerlstrom-Hultqvist et al., 2010, Morrison et al., 2007, Wielinga et al., 2015). Results of comparative analysis have shown significant differences among the three genotypes in genome size, gene content, gene arrangement, nucleotide sequences, and surface molecule repertoires. For example, the genomes of AI and B have only about 70% nucleotide sequence identity across the syntenic regions (Adam et al., 2013, Franzen et al., 2009). The divergence between sub-assemblages AI and AII is approximately 1% in sequences, with differences mainly localized to the variable regions of the genomes. There are approximately 35 AII-specific genes but only two AI-specific genes (Ankarklev et al., 2015).

An approach has been developed recently for whole genome sequencing of *G. duodenalis* in clinical specimens (Hanevik et al., 2015). It uses a procedure similar to the one developed for *Cryptosporidium* spp. for isolating *G. duodenalis* cysts in clinical specimens. This could facilitate WGS-based characterization of *G. duodenalis* specimens and increase the use of comparative genomics in the identification of contamination source during giardiasis outbreaks, as indicated by the retrospective investigation of a drinking water-associated outbreak in Creston, British Columbia, Canada (Prystajecky et al., 2015).

## 6 Use of molecular diagnostic tools in epidemiological studies of *Cryptosporidium* spp. and *G. duodenalis* in USA

The use of molecular diagnostic tools in epidemiologic studies has significantly improved our understanding of the transmission of waterborne protozoan parasites in the United States, especially *Cryptosporidium* spp. Compared with other industrialized nations (Caccio and Chalmers, 2016, Learmonth et al., 2004, Nazemalhosseini-Mojarad et al., 2012, Ng et al., 2010, Waldron et al., 2011), the United States has a high diversity of *Cryptosporidium* spp. in humans at both species and subtype levels. In addition to *C. hominis* and *C. parvum*, several emerging *Cryptosporidium* species or genotypes, such as *C. ubiquitum* and chipmunk genotype I, are responsible for substantial numbers of cryptosporidiosis cases, especially in rural states (Feltus et al., 2006, Guo et al., 2015a, Li et al., 2014). For *C. ubiquitum*, humans in the United States are infected with rodent-adapted subtype families XIIb–XIId, whereas those in the United Kingdom are mainly with ruminant-adapted subtype family XIIa (Li et al., 2014). As *C. ubiquitum* is one of the most common *Cryptosporidium* species in drinking source water in the United States (Jiang et al., 2005, Yang et al., 2008) and rodent-adapted subtype families are also the dominant *C. ubiquitum* subtypes in drinking source water, consumption of drinking water contaminated by infected wildlife could be the source of human infections (Li et al., 2014). The genetic similarity in *Cryptosporidium* chipmunk genotype I among isolates from humans, wild mammals, and drinking source water supports the potential role of wildlife and drinking source water in the transmission of emerging *Cryptosporidium* spp. in rural United States (Guo et al., 2015a).

Genotyping and subtyping data on *C. parvum* occurrence in sporadic cases of cryptosporidiosis indicate that farm animals, especially pre-weaned calves, are important in cryptosporidiosis epidemiology in rural United States. Like in the United Kingdom (Chalmers et al., 2011b), there are apparent differences in the distribution of *C. hominis* and *C. parvum* between rural states and other states, with *C. parvum* as the dominant species in sporadic cases in Minnesota and Wisconsin and *C. hominis* as the dominant species in southern states (Feltus et al., 2006, Herges et al., 2012, Xiao et al., 2009). A high *C. parvum* subtype diversity was identified in humans in Minnesota and Wisconsin (Feltus et al., 2006, Herges et al., 2012). As the dominant subtypes are also common ones in pre-weaned calves in the United States (Xiao et al., 2007), these animals are potential sources of zoonotic infections in humans. This is also supported by the numerous reports of calf-contact associated outbreaks of human cryptosporidiosis in the United States and results of case-control studies (Xiao and Feng, 2008), and by results of MLFT analysis of *C. parvum* isolates from humans and cattle in Upper Midwest states (Herges et al., 2012). The recent identification of cryptosporidiosis occurrence in Nebraska due to occupational animal exposure, especially to cattle, reinforces the importance of zoonotic infection in cryptosporidiosis epidemiology in rural U.S. states (Su et al., 2017).

Among the > 20 *C. parvum* subtypes found in humans in the United States, IIaA15G2R1, is the dominant *C. parvum* subtype, responsible for numerous outbreaks of cryptosporidiosis (Table 5). Data from a recent MLST study have shown extensive heterogeneity within IIaA15G2R1. Because of the widespread occurrence of genetic recombination within the subtype, there is frequent discordance in subtyping results between *gp60* and other genetic markers. As a result, there is no clear population diversion between IIaA15G2R and non-IIaA15G2R subtypes (Feng et al., 2013). Other population differentiation exists within *C. parvum*, evident by the presence of one subpopulation with an epidemic population structure and wide geographic distribution and another subpopulation with a clonal population structure and geographic segregation. Genetic recombination between the epidemic and geographically segregated *C. parvum* populations appears to be associated with the emergence of the hyper-transmissible IIaA15G2R1 subtype, which is also the dominant *C. parvum* subtype in humans in other industrialized nations (Xiao, 2010). Thus, the IIaA15G2R1 subtype at the *gp60* locus is probably a fitness marker for *C. parvum* and the wide spread of IIaA15G2R1 subtype around the world appears independent of sequence characteristics at other genetic loci (Feng et al., 2013). This needs confirmations from comparative genomic analysis of WGS data generated in recent years.

**Table 5:** *Cryptosporidium hominis* and *Cryptosporidium parvum* subtypes in U.S. cryptosporidiosis outbreaks investigated by the Centers for Disease Control and Prevention.

Species	Subtype family	Subtype	Outbreak
*C. hominis*	Ib	IbA10G2	WI (1993), NV (1994), GA (1995), Washington, DC (2000), TX (2002), NY (2005), IL (2006) CO (2006), SC (2010)
		IbA9G3	FL (1995), OH (2003), NC (2013)
	Ia	IaA14R3	IL (2001)
		IaA15R3	NM-1 (2008), Dallas, TX (2008)
		IaA20R3	NM-1 (2008), NM-2 (2008), T (2008)
		IaA24R4	SC (2000), NE (2000)
		IaA28R4	OH (2005), SC (2006), PA (2007), ID (2007), AZ (2008), NM (2008), OH (2008), TX (2008), MO/IL (2010), CO (2011), IA (2014)
	Id	IdA14	OH (2006)
		IdA15G1	OH (2004), OK (2007), KS (2007), TX (2008)
		IdA16	Puerto Rico (2007)
		IdA17	KS (2003)
		IdA17G1	OH (2000)
		IdA18	WA (1997)
		IdA19	OH (2016)
	If	IfA12G1	OR (2009), WI-1 (2013), WI-2 (2013), FL-1 (2014), FL-2 (2014), GA-1 (2014), GA-2 (2014), FL (2015), TN-1 (2015), TN-2 (2015), VA (2015), AL (2016), AZ (2016)
	Ig	IgA20	HI (2015)
		IgA27	TX (2008)
*C. parvum*	IIa	IIaA15G2R1	ME (1993), MN (1997); OH (2003), NC (2011), NY (2011), Oregon (2011), NC (2011), WI (2011), MI (2012), NE (2012), OR (2013), ME (2014), VA (2015), FL (2016), ME (2016)
		IIaA15G2R2	IA (2013)
		IIaA16G1R1b	FL (2006)
		IIaA16G3R1	ID (2014)
		IIaA16G1R2	NC (2010)
		IIaA17G2R1	OH (2003), NC (2009), WI (2013)
		IIaA17G2R2	OK (2007), IL (2015)
		IIaA17G2R3	NC (2013)
		IIaA18G3R1	NM (2016)
		IIaA19G2R1	CO (2014)
		IIaA20G2R1	MS (2007)
	IIc	IIcA5G3	CO (2003), (2004)

A high genetic heterogeneity is also present in *C. hominis* in the United States. In addition to the virulent IbA10G2 subtype, which is the dominant *C. hominis* subtype in European countries and Australia (Ng-Hublin et al., 2017, Waldron et al., 2011), several other *C. hominis* subtypes are common in sporadic and outbreaks cases in the United States, such as IaA28R4 and IfA12G1R5 (Hlavsa et al., 2017, Xiao et al., 2009) (Table 5). In early years, IbA10G2 was the dominant *C. hominis* subtype in the United States. A new subtype, IaA28R4, however, appeared in the United States in 2005 and by 2007 it had become the dominant *C. hominis* subtype for sporadic cases and outbreaks in the United States (Feng et al., 2014, Xiao et al., 2009) (Table 5). Accompanied with the occurrence of the new subtype was a near three-fold increase in the incidence of cryptosporidiosis in the United States during 2004 to 2008 (Yoder and Beach, 2007, Yoder et al., 2010). In recent years, subtype IaA28R4 has been replaced by IfA12G1R5, which was first seen in AIDS patients and was responsible for a 2009 cryptosporidiosis outbreak associated with the care of an AIDS patient in Oregon. Since 2013, it has emerged as the dominant *C. hominis* subtype in sporadic and outbreak-associated cases (Hlavsa et al., 2017). During this period, the incidence of cryptosporidiosis in the United States has remained at a high level (CDC, 2016, Adams et al., 2015, Adams et al., 2016, Yoder et al., 2012).

Recent MLST and comparative genomic analysis indicate that genetic recombination has played a major role in the emergence of hyper-transmissible *C. hominis* subtypes in the United States. Results of the MLST analysis indicate that there were at least two origins of IaA28R4 within the United States. Like IIaA15G2R1 within the United States, discordant subtyping results of IaA28R4 isolates were observed between *gp60* and other genetic markers, indicating that the emergence of these IaA28R4 variants was largely due to genetic recombination (Feng et al., 2014). Although IaA28R4 was first identified in a cryptosporidiosis outbreak in Ohio in 2005 and was the cause of another outbreak in the same area in 2008, IaA28R4 circulating in Ohio was genetically different from IaA28R4 in southwestern states. Results of comparative genomic analysis indicate that the genomes of two IaA28R4 isolates were very similar to those of IbA10G2, indicating that IaA28R4 could be the progeny of genetic recombination of IbA10G2 and another *C. hominis* subtype of the Ia subtype family. The genetic recombination was most obvious in the subtelomeric and *gp60* regions of chromosome 6 (Guo et al., 2015c). Thus, genetic recombination might lead to the emergence of hyper-transmissible *gp60* subtypes of both *C. parvum* and *C. hominis*.

Molecular characterizations of animal and human isolates have also played a role in clarifications of the potential role of beavers in the transmission of *G. duodenalis* in the United States. Giardiasis has been popularly known as beaver fever in North America, probably because some hikers and campers became ill from drinking stream water presumably contaminated with *Giardia* cysts from beavers (Faubert, 1988). This was supported by results of investigations of several waterborne outbreaks of giardiasis in late 1970s and early 1980s, in which beavers in drinking source watershed were found to be shedding *G. duodenalis* cysts (Xiao and Fayer, 2008). Results of molecular characterizations have shown that beavers are frequently infected with *G. duodenalis* assemblage B, which is the major cause of giardiasis outbreaks in the United States. In studies conducted in Maryland and Massachusetts, 11 beavers were identified having assemblage B (Fayer et al., 2006, Sulaiman et al., 2003). In one drinking water-associated outbreak of giardiasis in New Hampshire, the assemblage B subtype found in human cases was different from known subtypes in humans, but identical to the one previously found in beavers in Massachusetts (Daly et al., 2010). Although these reports did not provide any concrete evidence of the initial contamination source, they suggest that beavers could be environmental reservoirs for sustaining the contamination of drinking source water by *G. duodenalis* assemblage B. This has been supported by the investigation of one waterborne giardiasis outbreak in Creston, British Columbia, in which one beaver was identified by epidemiologic investigations as the likely cause of source water contamination. WGS analysis of one beaver isolate and one human isolate from the outbreak generated very similar genomes of sub-assemblage AI (Prystajecky et al., 2015).

## 7 Conclusions and perspectives

The use of molecular diagnostic tools has significantly improved our understanding of cryptosporidiosis epidemiology. It has led to the identification of major differences in infection sources of *Cryptosporidium* spp. in humans between developing countries and industrialized nations, differences in clinical presentations and virulence among *Cryptosporidium* species and *C. hominis* subtypes, and roles of genetic recombination in the emergence of virulent and hyper-transmissible *C. hominis* and *C. parvum* subtypes. These tools are now widely used in outbreak investigations and surveillance of cryptosporidiosis in industrialized nations (Chalmers and Caccio, 2016, Hlavsa et al., 2017). With the introduction of whole genome sequencing and comparative genomics in *Cryptosporidium* characterization, it is expected that molecular epidemiology of cryptosporidiosis would remain a highly active research area.

Despite these major achievements, many challenges remain. After over 20 years of major investments in drinking water treatment as a response to the massive 1993 Milwaukee outbreak, cryptosporidiosis remains the most important waterborne disease in industrialized nations (Efstratiou et al., 2017), incidence of cryptosporidiosis in the United States has increased nearly threefold since 2005 (Painter et al., 2016), and drinking water-associated outbreaks of cryptosporidiosis still present a significant public health and economic problem in European countries (Caccio and Chalmers, 2016, Chyzheuskaya et al., 2017). The identification of environmental and epidemiological factors involved requires systematic molecular characterizations of *Cryptosporidium* specimens submitted to national cryptosporidiosis surveillance systems. The latter has been in place for some time in the United Kingdom (Chalmers et al., 2011b) but is in the early stage of implementation in the United States as part of the CryptoNet program (Hlavsa et al., 2017). While genotype and subtype data are useful in the identification and investigation of zoonotic outbreaks due to unusual *C. parvum* subtypes and differentiation of autochthonous and travel-associated *C. hominis* infections in European countries, their utilities are limited in molecular surveillance of the dominant *C. hominis* subtypes in Europe (IbA10G2) and the United States (currently IfA12G1R5). This was demonstrated in the investigation of an increased transmission of IbA10G2 in multiple European countries in late summer, 2012, in which molecular characterizations did not identify an endemic source (Fournet et al., 2013, Roelfsema et al., 2016). In fact, advanced molecular analysis beyond *gp60* sequence analysis would not increase significantly the discrimination of these hyper-transmissible *C. hominis* subtypes, including comparative genomic analysis (Chalmers and Caccio, 2016, Roelfsema et al., 2016). Supplemental epidemiological data are helpful in the investigation of such outbreaks.

Molecular analysis probably can exert its biggest impact on improving knowledge of cryptosporidiosis epidemiology in developing countries, where cryptosporidiosis has been identified as the second most cause of modest to severe diarrhea and diarrhea-associated mortality in young children (G. B. D. Diarrhoeal Diseases Collaborators, 2017, Kotloff et al., 2013, Platts-Mills et al., 2015), and diverse *C. hominis* subtypes are circulating in endemic areas (Squire and Ryan, 2017, Xiao, 2010). While data from genotyping and subtyping studies of clinical specimens have suggested that anthroponotic infection is important in cryptosporidiosis epidemiology in these areas, the role of various transmission routes is not very clear for the formulation of evidence-based intervention strategies. This would require the integrated use of molecular diagnostic tools in well-designed epidemiological studies, which usually involve the use of longitudinal cohort or case-control design, or regression and multivariate analyses of risk factors. Thus far, such studies are few in numbers (Adamu et al., 2014, Ajjampur et al., 2010, Akinbo et al., 2010, Cama et al., 2008, Cama et al., 2007, Eibach et al., 2015, Kattula et al., 2017, Korpe et al., 2016, Tumwine et al., 2005, Wumba et al., 2012), and WGS and comparative genomic analyses have not been used in characterizations of *Cryptosporidium* spp. in developing countries. The population genetics of *C. hominis* in developing countries is probably complicated; genetic recombination among diverse subtypes could occur more frequently as a result of high cryptosporidiosis endemicity. Advanced molecular characterizations of specimens from well-designed epidemiologic investigations will likely lead to better understanding of the spread of virulent *C. hominis* subtypes in under-developed communities, as demonstrated in a MLST characterization of *C. hominis* from a longitudinal cohort study of cryptosporidiosis conducted in a shantytown in Lima, Peru (Li et al., 2013).

In contrast, molecular analysis of *G. duodenalis* has thus far mostly generated data useful in understanding of zoonotic potential of isolates in various animals. Because of host-adaptation at both genotype and subtype levels, zoonotic transmission of *G. duodenalis* is likely to be less important in giardiasis epidemiology compared with cryptosporidiosis (Feng and Xiao, 2011, Ryan and Caccio, 2013). Much of the current attentions on zoonotic transmission of *G. duodenalis* have focused on assemblage A because of its wide occurrence in animals compared with assemblage B. As humans are more commonly infected with assemblage B and there is a difference in the distribution of assemblage A subtypes between humans and animals, more attention should be directed to zoonotic transmission of assemblage B, which appears to be to the dominant *G. duodenalis* genotype in a few groups of animals, such as beavers, muskrats, chinchillas, rabbits, horses, and nonhuman primates (Debenham et al., 2017, Karim et al., 2015, Karim et al., 2014, Pantchev et al., 2014, Qi et al., 2015a, Qi et al., 2015b, Santin et al., 2013, Sricharern et al., 2016, Sulaiman et al., 2003, Traub et al., 2005, Traversa et al., 2012, Ye et al., 2012, Zhang et al., 2012). Some of the animals are aquatic or their fecal materials can be easily transported to water, thus could contribute to source water contamination with *G. duodenalis* assemblage B. The identification of assemblage E humans in several recent studies indicates that it could be an emerging human pathogenic *G. duodenalis* genotype, even in urban areas (Abdel-Moein and Saeed, 2016, Fantinatti et al., 2016, Scalia et al., 2016, Zahedi et al., 2017). Additional molecular epidemiological investigations should be conducted to identify its source and transmission routes.

The use of molecular diagnostic tools has unfortunately not improved significantly our knowledge of endemic and epidemic giardiasis in humans. This is due to both technical difficulties and neglect. Thus far, most molecular epidemiological studies of giardiasis in humans are conducted with clinical specimens from outpatients. The long duration of cyst shedding after the initial infection, presence of allelic sequence heterozygosity in some genotyping and subtyping markers, high occurrence of concurrent infections with mixed genotypes, and multiple episodes of infections in early childhood have made it challenging to delineate the transmission mechanism and virulence of *G. duodenalis* in humans. With recent developments in whole genome sequencing and comparative genomic analysis of *G. duodenalis*, new genotyping and subtyping markers can be identified for the development of next generation molecular tools (Ankarklev et al., 2015, Wielinga et al., 2015). As assemblages A and B evolve very differently and have divergent genomes (Adam et al., 2013, Franzen et al., 2009), it may be useful to develop assemblage-specific subtyping tools. The use of these advanced molecular diagnostic tools in epidemiological investigations of endemic giardiasis in developing countries and in surveillance and outbreak investigations of epidemic giardiasis in industrialized nations, as performed in several recent studies (Jerez Puebla et al., 2015, Minetti et al., 2015b, Pijnacker et al., 2016), may lead to improved understanding of molecular epidemiology of giardiasis.
